# A case management report: a collaborative perioperative surgical home paradigm and the reduction of total joint arthroplasty readmissions

**DOI:** 10.1186/s13741-016-0051-2

**Published:** 2016-10-18

**Authors:** Navid Alem, Joseph Rinehart, Brian Lee, Doug Merrill, Safa Sobhanie, Kyle Ahn, Ran Schwarzkopf, Maxime Cannesson, Zeev Kain

**Affiliations:** 1 Department of Anesthesiology & Perioperative Care, School of Medicine, University of California, Irvine, 333 City Boulevard West Side, Orange, CA 92868-3301 USA; 2 Division of Adult Reconstruction, Department of Orthopedic Surgery, NYU Langone Medical Center, Hospital For Joint Diseases, New York, USA; 3 Department of Anesthesiology & Perioperative Care, School of Medicine, University of California, Los Angeles, Los Angeles, USA; 4 Center for Stress & Health & Department of Anesthesiology & Perioperative Care, School of Medicine, University of California, Irvine, Orange, USA

**Keywords:** Anesthesia, Perioperative surgical home (PSH), Surgical readmissions, Perioperative medicine, Readmission reduction, Hospital discharge, Total joint arthroplasty (TJA)

## Abstract

**Background:**

Efforts to mitigate costs while improving surgical care quality have received much scrutiny. This includes the challenging issue of readmission subsequent to hospital discharge. Initiatives attempting to preclude readmission after surgery require planned and unified efforts extending throughout the perioperative continuum. Patient optimization prior to discharge, enhanced disease monitoring, and seamless coordination of care between hospitals and community providers is integral to this process. The perioperative surgical home (PSH) has been proposed as a model to improve the delivery of perioperative healthcare via patient-centered risk stratification strategies that emphasize value and evidence-based processes.

**Results:**

This case report seeks to specifically describe implementation of readmission reduction strategies via a PSH paradigm during total joint arthroplasty (TJA) procedures at the University of California Irvine (UCI) Health. An orthopedic surgeon open to collaborate within a PSH paradigm for TJA procedures was recruited to UCI Health in October of 2012. Institution specific data was then prospectively collected for 2 years post implementation of the novel program. A total of 328 unilateral, elective primary TJA (120 hip, 208 knee) procedures were collectively performed. Demographic analysis reveals the following: mean age of 64 ± 12; BMI of 28.5 ± 6.2; ASA Score distribution of 0.3 % class 1, 23 % class 2, 72 % class 3, and 4.3 % class 4; and 62.5 % female patients. In all, a 30-day unplanned readmission rate of 2.1 % (95 % CI 0.4–3.8) was observed during the study period. As a limitation of this case report, this reported rate does not reflect readmissions that may have occurred at facilities outside UCI Health.

**Conclusions:**

As healthcare evolves to emphasize value over volume, it is integral to invest efforts in longitudinal patient outcomes including patient disposition subsequent to hospital discharge. As outlined by this case management report, the PSH provides an institution-led means to implement a series of care initiatives that optimize the important metric of readmission following TJA, potentially adding further value to patients, surgical colleagues, and health systems.

## Background

Repeat admission after hospital discharge remains a significant and complex problem (Joynt and Jha 2012; Lucas and Pawlik 2014; Allaudeen et al. 2011; Merkow et al. 2015; Garrison et al. 2013; Zmistowski et al. 2013; Saucedo et al. 2014). Nearly one in every five patients is readmitted within 30 days of hospital discharge, accounting for an estimated $15 billion in healthcare spending annually (Allaudeen et al. 2011). This alarmingly high rate of unplanned readmission and the associated costs are both unsustainable and unacceptable. As the Affordable Care Act and other efforts to reduce the cost of healthcare are assimilated into payer policies, there is urgency for the healthcare industry to implement collaborative care models that emphasize value over volume (Ho and Sandy 2014; Szokol and Stead 2014; Schroeder and Frist 2013; Hertzberg 2013). Accountable care organizations (ACOs) are rapidly proliferating and can be defined as an integrated group motivated to provide enhanced patient care at a reduced cost for a defined population of patients (Barnes et al. 2014; Decamp et al. 2014; Epstein et al. 2014).

The Centers for Medicare & Medicaid Services (CMS) established the Hospital Readmissions Reduction Program in 2013.^1^ Under this program, payments are now reduced for hospitals with 30-day readmission rates higher than a national benchmark for patients with the diagnoses of heart attack, heart failure, or pneumonia. Payment reduction is expanding and now includes readmission after surgical procedures (specifically elective total hip or total knee arthroplasty and coronary artery bypass graft surgery). CMS has also begun to associate 30-day readmission rates after elective total joint arthroplasty (TJA) procedures as an overall surrogate measure of hospital quality (Grosso et al. 2012). Payers, providers, and policymakers have much impetus to enhance the quality of patient care during TJA procedures while reducing expenditures (Bozic et al. 2014).

The perioperative surgical home (PSH) has been proposed as a model to improve the delivery of perioperative healthcare via patient-centered optimization strategies that involve risk stratification and standardization of care (Kash et al. 2014; Cyriac et al. 2016; Raphael et al. 2014; Garson et al. 2014; Cannesson et al. 2014; Schweitzer et al. 2013; Mackey and Schweitzer 2014; Vetter et al. 2013, 2014; Desebbe et al. 2016). The PSH also introduces clinical opportunities for varied providers to collectively enhance care of the surgical patient (Kash et al. 2014). A prime example is the reduction of surgical readmissions, as in theory this would yield improved longitudinal care at reduced costs (Joynt and Jha 2012). As such, this case report will outline one model of a collaborative perioperative team operating within a PSH practice-model to reduce surgical readmissions after TJA procedures.

## Methods

### Implementation of a perioperative surgical home for total joint arthroplasty (TJA) procedures

With unique and cumulative insights, a multitude of disciplines including anesthesiology, orthopedic surgery, nursing, pharmacy, case management, social work, nutrition, physical therapy, and information technology closely collaborated to institute a PSH for primary TJA (hip and knee) procedures at UCI Health in October of 2012 (Cyriac et al. 2016; Raphael et al. 2014; Garson et al. 2014). Weekly meetings were coordinated and LEAN Six Sigma methodology (De Koning et al. 2006) was used to ultimately manifest clinical pathways that paralleled “patient-centered, multidisciplinary, and integrated care (Grocott and Mythen 2015)” as opposed to fragmented, variable, and inefficient care (Mackey 2012; Berwick and Hackbarth 2012). As an integral component of the implemented TJA PSH paradigm, concerted strategies designed to avert post-surgical readmissions were employed at all phases encountered during the perioperative continuum.

### Preoperative measures to optimize readmission risk

**Fig. 1:**
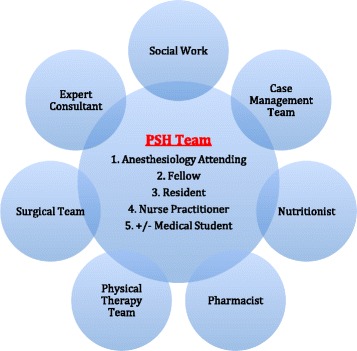
Members of the rounding PSH team dynamically work in concert with other key providers to proactively preclude factors that may contribute to a readmission. Note: The fellow is an anesthesiology graduate conducting a perioperative medicine fellowship and the resident is an anesthesiology resident conducting an innovative PSH rotation

**Fig. 2:**
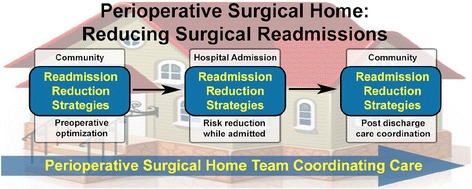
The PSH team strives for continuous care transitions between the community and hospital period with relevant information clearly relayed

The Center for Perioperative Care (CPC) at UCI Health took the role of closely working with the Case Management team before surgery to ensure that longitudinal patient disposition was planned as early as possible, long before admission. Factors that contribute to an unplanned readmission were proactively confronted. For example, transportation needs were assessed and durable medical equipment arrangements were made at the time that a surgery date was scheduled. Moreover, “preferred” pharmacies, rehabilitation services, and skilled nursing facilities were identified with the patient and family. Financial arrangements were not made, and patients maintained selection autonomy. However, the term “preferred” denoted that the case management, surgery, and anesthesiology teams met with these providers and outlined post-hospital (discharge) protocols, goals and expectations as outlined by the tailored PSH clinical care pathways (Kash et al. 2014; Cyriac et al. 2016; Raphael et al. 2014; Garson et al. 2014; Vetter et al. 2013; Desebbe et al. 2016). Another important role for the CPC included the accurate identification of the patient’s primary care provider (PCP) and specialists such as chronic pain providers. This allowed for the PSH team (Fig. 1) to play a role as the liaison that manages care transitions between the community and hospital period, aspiring to achieve a seamless “handshake” between the two (Fig. 2). Transitions or “handoffs” are particularly vulnerable exchange points that expose patients to lapses in quality and safety (Naylor et al. 2011; Auerbach et al. 2016). Lastly, the CPC clinic provided educational classes that both managed patient expectations and elucidated important safety initiatives. An important point is that the specific nature of the patient formed the center of the care model, rather than the diagnosis or planned procedure, a shift in focus that was significant in improving the quality and value of care (Brummett and Clauw 2015).

### Postoperative measures to optimize readmission risk

**Fig. 3:**
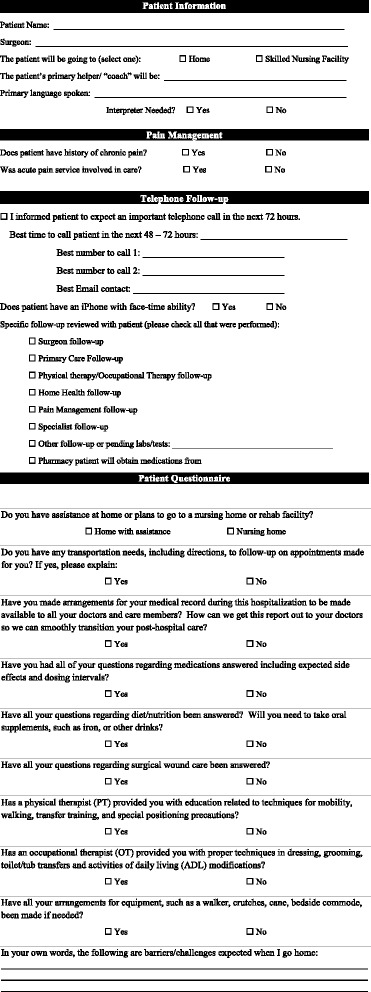
Discharge readiness checklist to be reviewed with the patient by the PSH team prior to discharge

Postoperatively, a collaborative PSH team longitudinally followed all enrolled PSH patients until the day of discharge. Leveraging evidence-based medicine and technology, care that transpired after the surgical intervention was managed for discharge optimization. This included providing fulltime coverage for a diverse array of post-surgical patients, often with multiple medical comorbidities. Goals included enhancement of discharge processes by continually working with other key disciplines (Fig. 1) and the proactive identification and confrontation of factors known to contribute to a readmission after surgery (Table 1). As a final step, a discharge readiness checklist was created as a tool for review by the PSH team with the patient before a discharge ensues (Fig. 3).

**Table 1:** Most common risk factors and causes that contribute to readmission risk after a surgical intervention

Risk factors (Lucas and Pawlik 2014)	Causes (Merkow et al. 2015)
Multiple comorbidities	Surgical site infection
Long length of hospital stay	Ileus
Postoperative complications	Postoperative bleeding

### Post-discharge measures to optimize readmission risk

**Fig. 4:**
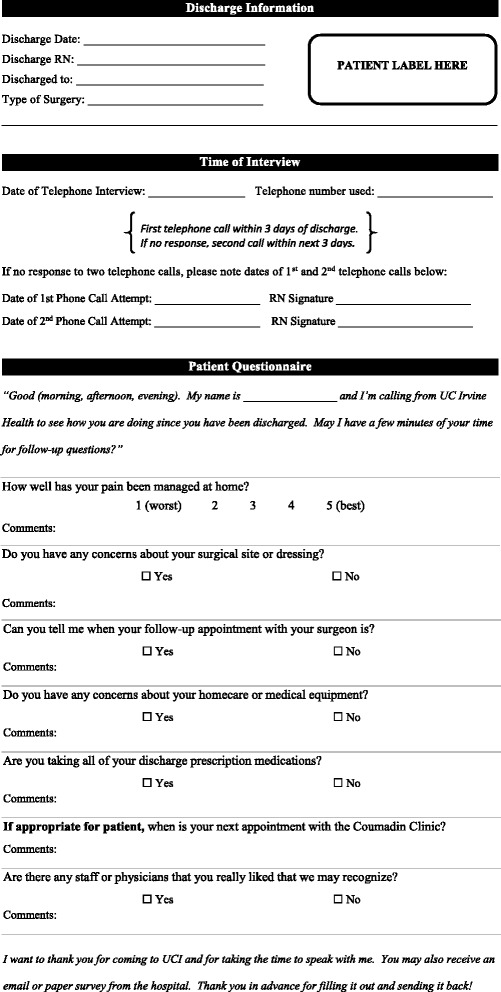
Standardized list of post-discharge questions during nurse follow-up calls

**Fig. 5:**
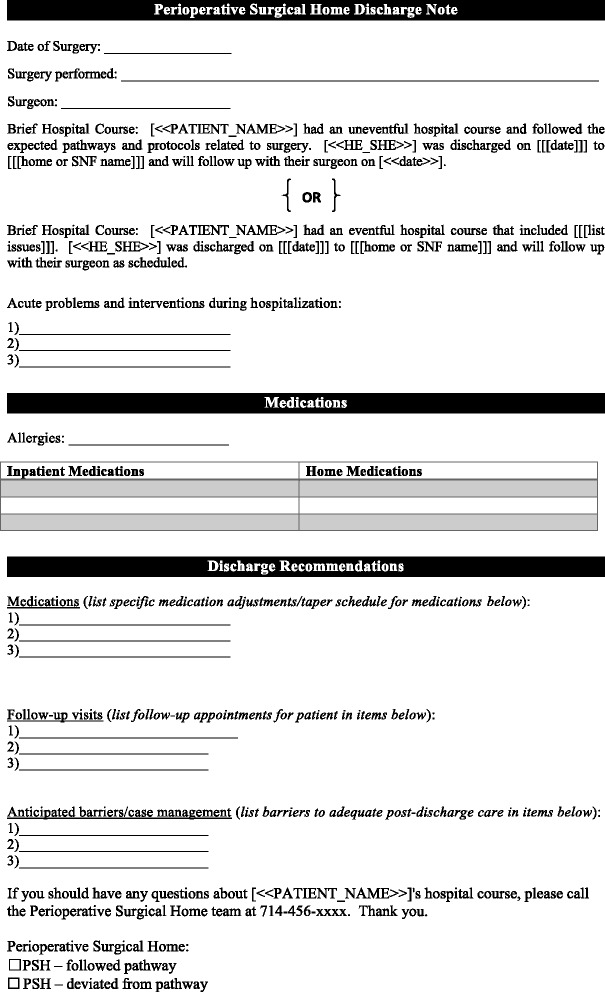
This standardized discharge note prepared by the PSH team is replete with information regarding the patient’s perioperative medical care. It is integrated into the electronic medical record and sent to the patient’s community primary care provider on the day of discharge

The post-discharge period was a critical time to continue guiding a patient to enhanced recovery. A phone call was made by designated inpatient orthopedic nursing staff to all patients within 72 h of discharge to assure that discharge milestones were being met appropriately. The simple standardized list of questions was scripted in advance as a component of the PSH clinical pathway and integrated into the electronic medical record (Fig. 4). While the majority of calls were uneventful, triage occurred when answers indicated that an intervention may be required. Further measures taken to ensure that post-discharge care was not fragmented included sending a PSH note replete with information regarding the patient’s perioperative medical care to the patient’s PCP at the time of discharge (Fig. 5). To further bolster the transition in care, the PSH team supplemented with planned phone calls to the PCP and/or specialist provider for all high-risk patients with perioperative complications.

In addition, when emergency care was needed, all program enrolled patients were instructed to return to our own institution when feasible. When a PSH patient presented to the emergency room within 30 days of discharge, an automated page was immediately sent to the PSH team for the opportunity to contribute a value-added (Hertzberg 2013) assessment and care plan. Simultaneous with the patient’s presenting signs and symptoms, assessment was made, and appropriate steps were taken to intervene and help manage the patient as deemed appropriate. Efforts were made to collaborate with other specialists as indicated, and Table 2 specifically outlines some of the point of care opportunities at the patient’s bedside for an anesthesiologist to potentially avert an unnecessary readmission.

**Table 2:** Point of care (POC) assessment and intervention prospects to avert hospital readmissions

Opportunities to avert a readmission in the emergency room
1. Point of care (POC) ultrasonography (Ramsingh et al. 2015) for bedside assessment of cardiopulmonary function, volume status, vascular access, gastric volume, bladder volume
2. Advanced pain management intervention including multimodal therapy with regional techniques ± indwelling catheters
3. Liaisons to surgical services that may be confined to the operating room and delayed in patient assessment
4. Patient education, medication reconciliation, expectation management, multimodal anxiolysis
5. Postoperative nausea and emesis management
6. Assessment and management of perioperative medical complications
7. Assistance with transitions in care with community primary care providers (PCPs) or other specialists to provide rapid and appropriate disposition planning

### Results and Discussion

This report describes our findings for unplanned 30-day readmissions in the first 2 years of the novel PSH program (October 1 2012 until September 30 2014). Institutional Review Board (IRB) approval was obtained for prospective data collection, analysis, and reporting (IRB HS # 2012-9273). Data was corroborated using hospital-based decision support, electronic medical record (Allscripts, Chicago, IL), and AIMS (SIS, Alpharetta, GA). A total of 328 unilateral, primary, and elective TJA (120 hip, 208 knee) procedures were collectively performed in year 1 and year 2. Demographic analysis reveals the following: mean age of 64 ± 12; BMI of 28.5 ± 6.2; ASA Score distribution of 0.3 % class 1, 23 % class 2, 72 % class 3, and 4.3 % class 4; and 62.5 % female patients.

In all, a 30-day unplanned readmission rate of 2.1 % (95 % CI 0.4–3.8) was observed during the study period (Table 3) (Cyriac et al. 2016). During the 2-year study period, unplanned 30-day readmissions were noted to be due to variable etiologies, but surgically related complications such as dislocation or fracture of the prosthetic joint predominated (Table 3). The increased readmission rate observed in year 2 of the program (Table 3) is not attributable to dissimilar patient demographics or comorbidities (Cyriac et al. 2016) and is likely an incidental finding reflective of the small sample size. While the program protocol included approaches to send patients to our own institution for emergency care when possible, it should be emphasized that the reported readmission rates do not incorporate readmissions that potentially occurred beyond UCI Health.

**Table 3:** Post PSH implementation TJA and readmission data year 1 and year 2

	Year 1 post PSH implementation	Year 2 post PSH implementation	2-year cumulative
Total number of total joint arthroplasty	144	184	328
Total number of unplanned 30-day readmissions	1	6	7
Readmission diagnosis	• Disruption of external wound	• Dislocation of prosthetic joint • Malaise • Stress fracture of femoral neck • Peri-prosthetic fracture • Contracture of tendon • Acute renal failure	
30-day readmission rate^a^	0.7 %	3.3 %	2.1 %

^a^Institution specific

**Fig. 6:**
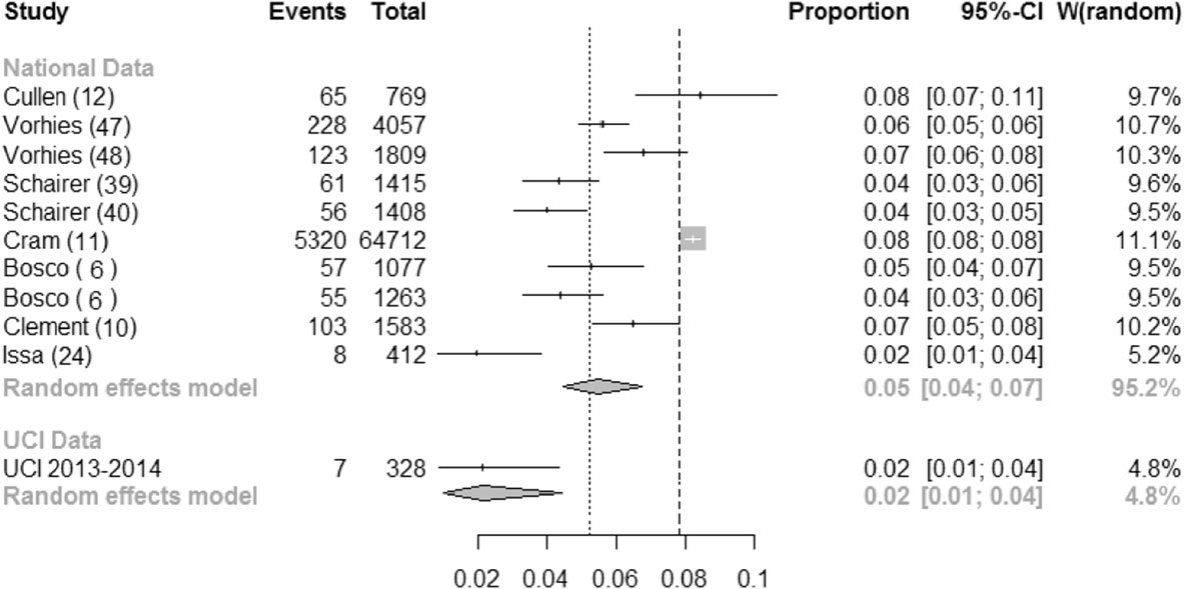
Meta-analysis of UCI readmission results in comparison to previously reported results. Forest plot and statistics for nine previously reported readmission rates in studies of TKA and THA patients and comparison to the UCI data set from 2013 to 2014. *CI* confidence interval, *W* weight of study in meta-analysis (Bosco et al. 2014; Clement et al. 2013; Cram et al. 2012; Cullen et al. 2006; Issa et al. 2014; Schairer et al. 2014a; Schairer et al. 2014b; Vorhies et al. 2011; Vorhies et al. 2012)

UCI Health did not have an established TJA program prior to 2012 to allow an unplanned readmission evaluation relative to an institutional baseline. As such, a comparison with previously published national results was considered to be useful. A systematic review and meta-analysis by Bernatz et al. (2015) listed the individual results of nine individual studies on readmission rates for TKA or THA nationally. A de novo meta-analysis of these nine studies reveals a total of 6076 readmissions in 78,505 patients—a 30-day unplanned readmission rate of 5.5 % (95 % CI 4.5–6.7) calculated by the inverse variance method using a random effects model, with significant heterogeneity between studies (*Q* = 145.5, *p* < 0.0001). For the meta-analysis, we used the statistical methodology of Bernatz et al. (2015) to analyze the same final data sample they used in their study, with the addition of our own data as a new group. When comparing these nine pooled results to our own results using the same meta-analytical method, we find the difference is significant at the 0.05 level (*Q* = 6.07; *p* = 0.014 for difference) (Fig. 6). Further comparison of our readmission data to a national benchmark rate of 4.6 % after TJA should also be noted.^2^ This reported national estimate is specific to Medicare beneficiaries and is again inclusive of unplanned readmission to an any acute care hospital within 30 days after discharge from a hospital.

## Conclusions

Preventable readmissions remain a common target for the improvement of healthcare (Joynt and Jha 2012; Lucas and Pawlik 2014; Allaudeen et al. 2011; Merkow et al. 2015; Garrison et al. 2013; Zmistowski et al. 2013; Saucedo et al. 2014; Jencks et al. 2009; Tsai et al. 2013; Joynt et al. 2011). Although surgical readmissions account for less than a quarter of all hospital readmissions (Jencks et al. 2009), analysis has revealed significant disparities in re-hospitalization rates after surgery between institutions (Lucas and Pawlik 2014; Tsai et al. 2013). It can be debated as to whether this appropriately parallels the quality of care rendered by a particular hospital or rather is a reflection of greater readmission risk for hospitals providing care to patient populations with greater disease burden or lower socioeconomic status and support (Tsai et al. 2013; Joynt et al. 2011). Regardless, a large review demonstrated that the majority of surgical readmissions are attributable to new complications that can be predicted and are characteristic of a particular procedure (Merkow et al. 2015). These findings suggest that appropriate risk stratification and thoroughly preparing patients for post-hospital care present significant potential for healthcare systems endeavoring to reduce surgical readmissions.

In this case management report, we outline the use of the PSH as a model to reduce the incidence of readmission after TJA surgery. Our model resulted in lower readmission rates than those reported nationally in a statistically significant manner. There are several limitations that should be noted, including a limited sample size and duration, lack of control group of patients not enrolled in the program, and the ability to only capture institution-specific readmissions. Nevertheless, we submit that understanding general risk factors and causes (Table 1) for readmission in surgical patient populations will facilitate the development of evidence-based models aimed at both optimizing patients for early discharge as well as decreasing preventable readmission. While there are certainly recurring factors that must be accounted for, efforts aimed at decreasing unplanned readmissions are ultimately much more complex and dynamic. Corrective efforts must be holistic and tailored to the patient, surgery, and the facility, as each readmission ultimately reflects multifactorial underpinnings. For instance, we learned that at our institution post-surgical joint dislocations and fractures were the primary culprits for unplanned readmissions (Table 3), and future pathway revisions will evolve to optimize patient education and physical therapy for fall prevention. A delicate balance must also be achieved for proper “discharge optimization,” as the inherent investment of time and resources required may be significant. Frank divergence exists between reducing readmission and other important hospital goals, such as a fast-track discharge (Kehlet and Wilmore 2005) and decreased length of stay (Pearson et al. 2001).

Pathways and systems that are integrated into discharge processes need thorough vetting and contribution from practitioners with diversified perspectives. The PSH provides an institution-led means to optimize patient care by unifying resources available throughout the perioperative continuum (Kash et al. 2014; Cyriac et al. 2016; Raphael et al. 2014; Garson et al. 2014; Cannesson et al. 2014; Schweitzer et al. 2013; Mackey and Schweitzer 2014; Vetter et al. 2013, 2014). Beginning with an indication for surgery and extending to the post-discharge transfer of care back to a PCP, there are an abundance of opportunities to incorporate the evidence-based initiatives of the PSH. By means of interdisciplinary discharge planning and oversight of process outcomes that re-compose variable practices into uniformly implemented evidence-based models, potential gaps in care that expose patients to harm or potential readmission can be minimized. As outlined by the Institute for Healthcare Improvement’s “Triple Aim,” much of healthcare reform has revolved around the multifaceted goals of improving patient satisfaction, while decreasing morbidity and costs of care (Vetter et al. 2014). With this in mind, it is important to continually search for ways to improve longitudinal patient outcomes as illustrated by this case report describing the potential impact of the PSH care model on the important metric of readmission following elective TJA surgery.

## Abbreviations

CMS: Centers for Medicare & Medicaid Services
CPC: Center for Perioperative Care
IRB: Institutional Review Board
PCP: Primary care provider
POC: Point of care
PSH: Perioperative surgical home
TJA: Total joint arthroplasty
UCI: University of California, Irvine

## Footnotes

1 Centers for Medicare & Medicaid Services. Readmission Reductions Program, 2014. Available from URL: https://www.cms.gov/medicare/medicare-fee-for-service-payment/acuteinpatientpps/readmissions-reduction-program.html, (Last Viewed June 2016)

2 Medicare.gov. 30-day unplanned readmission and death measures: complication rate for hip/knee replacement patients. 2016. Available from URL: https://www.medicare.gov/hospitalcompare/Data/30-day-measures.html, (Last Viewed August 2016)
